# Variation in the expression of ergot alkaloids between individual tillers of perennial ryegrass

**DOI:** 10.3389/fchem.2014.00107

**Published:** 2014-11-24

**Authors:** Wade J. Mace, Kristy L. Lunn, Navjot Kaur, Catherine M. Lloyd-West

**Affiliations:** Grasslands Research Centre, AgResearch LimitedPalmerston North, New Zealand

**Keywords:** ergot alkaloids, quantitation, *Lolium perenne*, endophyte, liquid chromatography-mass spectroscopy

## Abstract

*Epichloë* fungal endophytes of cool season grasses are well-known to produce a range of alkaloids of benefit to the host. Some of these compounds are advantageous to agriculture due to qualities that promote pasture persistence (e.g., the loline class of alkaloids confer insect protection) while others are detrimental to the well-being of grazing livestock. The ergot alkaloids (e.g., ergovaline), produced in ryegrass and tall fescue associations, causes poor animal health in farming regions in many countries around the world and further study is required to improve our knowledge on this class of compounds. Here we present the application of a quantitative LC-MS/MS (liquid chromatography coupled to mass spectrometry) method measuring eight ergot alkaloids (chanoclavine, agroclavine, elymoclavine, lysergol, lysergic acid, ergine, lysergyl-alanine, ergovaline) produced by endophyte infected grasses, to monitor levels in individual tillers from multiple plants of a single cultivar of perennial ryegrass (*Lolium perenne* cv. “Grasslands Samson”) infected with a common toxic endophyte strain (*Epichloë festucae* var. *lolii*). Monitoring the expression in individual tillers allows an estimation of the variability within a plant (between tillers) as well as between plants. The study showed that there is significant variation in the concentration of the ergot alkaloids between tillers of a single plant, at or exceeding the level of variation observed between individual plants of a population. This result emphasizes the fundamental importance of robust experimental design and sampling procedures when alkaloid expression assessment is required and these need to be rigorously tailored to the hypothesis being tested.

## Introduction

Fungal endophytes of cool season grasses, the *Epichloë* “endophytes,” are well-known for their production of bioactive alkaloids and the benefits these compounds confer to their plant hosts (Bush et al., 1997; Kuldau and Bacon, 2008). Aside from those alkaloids that protect the plant from insect herbivory (Breen, 1994), these endophytes also produce alkaloid toxins active against mammalian herbivores (Schmidt and Osborn, 1993) such as the indole diterpene, lolitrem B and the ergot alkaloid, ergovaline. In an agricultural context these toxins are undesirable due to their detrimental health effects to grazing animals and subsequent profitability of the farming enterprise (Hoveland, 1993).

Artificially created perennial ryegrass/endophyte associations, or novel associations, are a key factor in New Zealand's agri-business success (Johnson et al., 2013). This is due to the ability to create novel associations that are absent of the detrimental endophyte toxins (most importantly lolitrem B) and the associated animal ill-health while still possessing advantageous traits like insect deterrent compounds (such as peramine). Similarly, tall fescue/endophyte associations, which lack the production of ergovaline, are available in the USA (Bouton et al., 2002). But the dominance of the natural tall fescue/endophyte associations, which produces ergovaline, still has a significant impact on animal health in North American agriculture. Hence there has been (and continues to be) significant research into the effects of the ergot alkaloids (predominantly ergovaline) on animal health (Schmidt and Osborn, 1993), physiological response, mode of action, management practices that can mitigate animal health impacts, seasonal expression (Rogers et al., 2011), and distribution *in planta* (Spiering et al., 2002).

**Figure 1:**
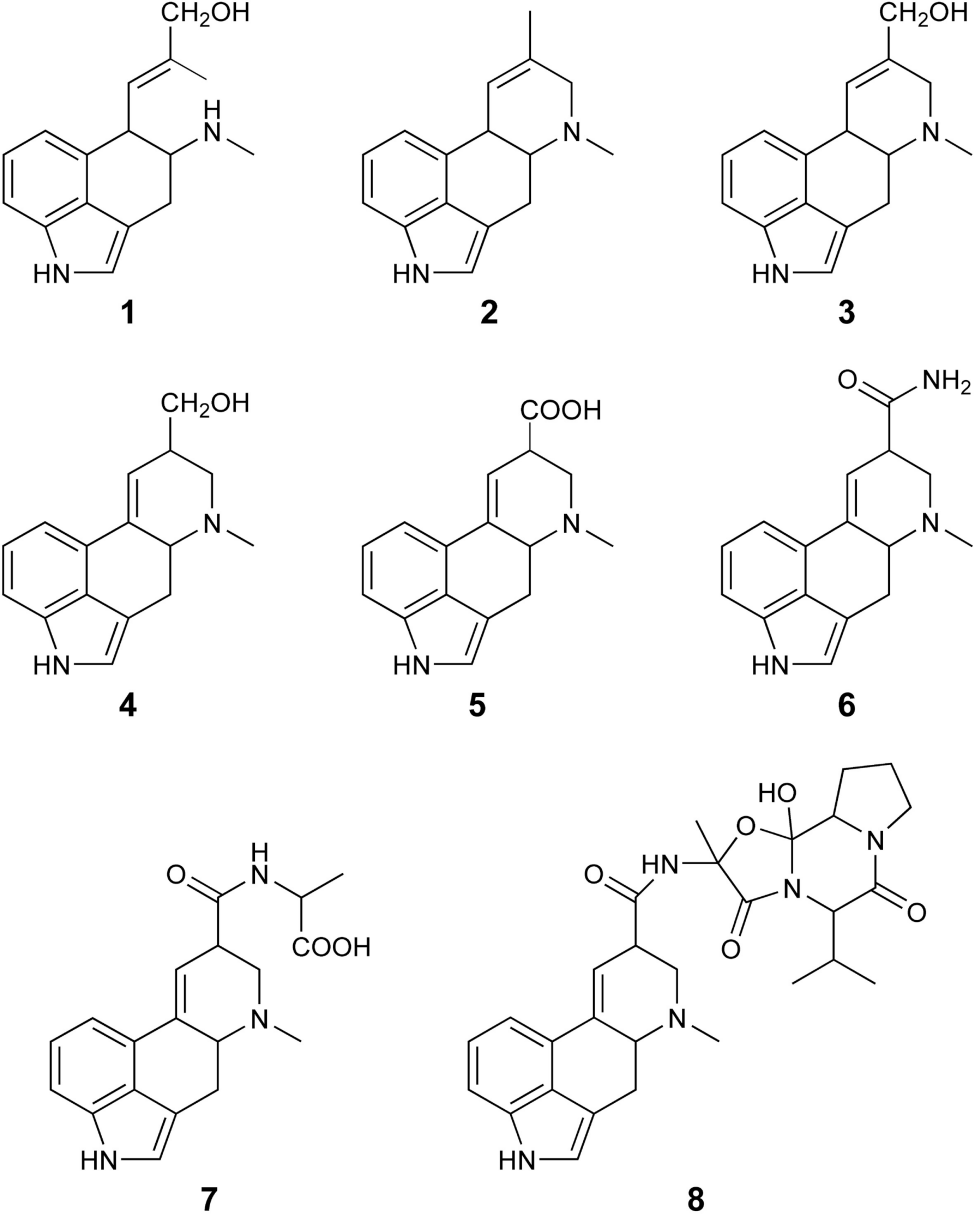
**Ergot alkaloids that can be detected in cool season grasses infected with *Epicloë* endophytes; chanoclavine (1), agroclavine (2), elymoclavine (3), lysergol (4), lysergic acid (5), ergine (6), lysergyl-alanine (7), and ergovaline (8)**.

While ergovaline has been the main focus, it is important to consider the other ergot alkaloids that are expressed with ergovaline in the various perennial ryegrass and tall fescue endophyte associations. Figure 1 shows the a range of the ergot alkaloids that can be detected in extracts of cool season grasses infected with *Epichloë* endophytes. Ergine and lysergyl-alanine are likely byproducts of the lysergyl peptide lactam intermediate (Panaccione et al., 2003), and as shown by the results of the current study, can comprise a significant proportion of the ergot alkaloids expression in perennial ryegrass/endophyte associations.

Research has been undertaken into the distribution of endophyte alkaloids between different plant structures. This has shown that there is a decreasing gradient of ergovaline concentration from the base to the tip of the tiller. However, many previous studies investigated population averages due to multiple samples from individual plants needing to be pooled to supply sufficient material for quantitative analyses. Similarly, research has been undertaken investigating the difference in expression levels between newly emerged leaves and older more mature plant structures, but again this research required the pooling of samples (Spiering et al., 2002, 2005). It has also been shown (Welty et al., 1994) that there are significant differences in expression between plants within a population with the expression difference a heritable trait (Easton et al., 2002). This factor has been exploited in agricultural systems to select for populations with higher or lower ergovaline expression (Adcock et al., 1997; Pennell et al., 2010).

To date no research has been undertaken on individual tillers looking at variation in expression within a single plant. If such variation exists, then a sampling regime for selecting high or low expressing populations would be critical, as would the sampling protocol for assessing any individual plant expression. The aim of the current research was to investigate the range of ergovaline (and selected ergot alkaloids) expression between individual tillers within a plant, and compare this to the range of expression observed between plants in a given population.

## Materials and methods

### Plant material

Seedlings of perennial ryegrass cv. Samson, infected with a common toxic strain of *Epichloë festucae* var. *lolii* [formally known as *Neotyphodium lolii* (Leuchtmann et al., 2014)] were raised in a glasshouse and their endophyte status assessed after 4 weeks from sowing seed (Simpson et al., 2012). Endophyte-free plants were discarded and endophyte-positive plants (25 individuals) were transferred to poly-bags (P3/4) with fresh potting mix. Plants were maintained in the glasshouse for approximately 14 months. Plant maintenance included routine watering and application of additional slow release fertilizer as required. Periodic trimming of plants with scissors to 20 mm above ground level to stimulate alkaloid production was conducted with a final trim to ground level conducted in February 2011 (late summer). 10 weeks after the final trim, six tillers were randomly selected from each plant and harvested to ground level, with the basal 40 mm of the tiller frozen, freeze-dried, and weighed. Individual tillers were milled using 2 × 3 mm stainless steel beads (5 m/s, 30 s) in 2 mL plastic vials (FastPrep FP120, Savant Instruments Inc., Farmingdale, NY, USA).

### Sample extraction

Tillers were extracted using a modification of previous methods (Mol et al., 2008; Rasmussen et al., 2012). Briefly; milled tiller samples were extracted with 500μL of 75% methanol (containing ergotamine (0.54μg/mL) as an internal standard) for 1 h in the dark. Samples were centrifuged (5000 g^1^, 5 min) and the supernatant transferred to 2 mL amber HPLC vials via a 0.45 μm syringe filter (PVDF).

### Ergot alkaloid analysis

Samples were analyzed according to Rasmussen et al. (2012), using a Thermo TSQ triple-quadrupole mass spectrometer equipped with an Accela 1250 HPLC system. Chromatography was achieved using a Kinetex XB-C18 column (100 × 2.1 mm, 2.6μ, Phenomenex). The method allows the quantification of chanoclavine (1), agroclavine (2), elymoclavine (3), lysergol (4), lysergic acid (5), ergine (6), lysergyl-alanine (7), ergovaline (8), and epimers thereof. Peak integration was conducted using LCQuan 2.7 (Thermo Fisher Scientific Inc., San Jose, CA, USA) with AgResearch in-house software used to determine alkaloid concentrations from peak areas and calculated standard curves.

### Data and statistical analysis

A weighted average of the individual tiller alkaloid concentrations was used to calculate a plant concentration for the purposes of comparing variation between plants. The formula $x_{plant}=\sum_{i\;=\;1}^{n}w_{i}x_{i}/\sum_{i\;=\;1}^{n}w_{i}$ describes the calculation, where *x_i_* = concentration of analyte *x* in tiller *i, w_i_* = weight of tiller *i*, and *x_plant_* = calculated concentration of analyte *x* in the plant.

Investigation of the proportions of variance between plant and within plant was preformed via a fully nested ANOVA of the alkaloid expression from individual tillers. A power analysis for determining the number of tillers that would be required to be harvested per plant was undertaken using a One-Way ANOVA model. Statistical analyses were conducted using Minitab® 16 (Minitab Pty Ltd, Sydney, Australia).

## Results

### Ergot alkaloid detection and quantitation

Not all alkaloids from the ergot alkaloid biosynthetic pathway were detected above the limit of quantitation; only chanoclavine, ergovaline, ergine, and lysergyl-alanine exceeded the limit of quantification (0.1 mg/kg *in planta*). Therefore, only these ergot alkaloids were used to evaluate intra- and inter-plant variation in alkaloid expression.

### Variation in ergot alkaloid expression

#### Variation between plants

There was a high level of variation in the expression of the detected alkaloids between plants in the population. Chanoclavine showed the least change in expression level (3 fold range between plants) compared to ergine which showed the highest with a greater than 25 fold range in expression. This was reflected in the coefficients of variation, where ergine showed the highest (71.3%) and chanoclavine the lowest (28.4%) (Table 1).

**Table 1:** **Summary statistics for the ergot alkaloid expression of the plants in the sample population; mean plant expression (mg/kg), minimum and maximum plant expression (mg/kg), coefficient of variation for plant expression**.

**Alkaloid**	**Chanoclavine**	**Ergovaline**	**Ergine**	**Lysergyl-alanine**
Mean	0.38	5.5	5.81	1.2
Min	0.18	0.65	0.14	0.47
Max	0.56	13.88	12.65	1.98
CV (%)	28.4	53.2	71.3	33.4

#### Variation within plants

Within the plants (between tillers of the same plant) there were also observations of a high level of variation in alkaloid expression. Coefficients of variation for any plant/alkaloid combination ranged from 8.3% to 90.8%. Table 2 shows the mean concentration and coefficient of variation for each plant/alkaloid combination.

**Table 2:** **Mean concentrations (mg/kg) (and coefficients of variation) of ergot alkaloids detected in tillers of individual plants of the population**.

**Plant**	**Chanoclavine**		**Ergovaline**		**Ergine**		**Lysergyl-alanine**	
	**Mean**	**CV (%)**	**Mean**	**CV (%)**	**Mean**	**CV (%)**	**Mean**	**CV (%)**
01	0.43	17.7	5.4	71.9	7.7	52.6	1.4	65.2
02	0.42	34.8	8.1	29.5	0.1	155.1	0.8	37.4
03	0.50	10.0	7.6	14.9	10.3	8.3	1.9	14.3
04	0.37	22.4	13.9	9.8	1.3	35.1	1.0	21.0
05	0.20	28.8	2.5	21.6	6.2	12.1	0.7	13.6
06	0.25	66.8	0.6	90.8	7.1	64.6	1.0	72.4
07	0.49	34.8	5.5	26.3	9.0	12.5	1.6	17.0
08	0.29	20.9	6.6	40.9	4.2	22.4	1.2	37.8
09	0.36	35.1	11.2	30.5	10.7	26.7	1.6	15.1
10	0.27	58.6	3.7	42.2	2.7	60.0	0.7	50.8
11	0.44	25.5	5.5	29.5	12.6	14.3	2.0	14.5
12	0.20	59.6	1.7	26.9	8.3	43.1	1.1	48.2
13	0.28	19.0	5.2	15.6	10.0	32.3	0.8	32.6
14	0.23	49.2	4.9	35.1	0.5	55.8	1.0	34.1
15	0.56	39.8	10.2	30.0	0.2	117.0	1.5	24.2
16	0.50	48.0	3.2	39.3	6.2	35.5	1.2	45.4
17	0.30	17.3	5.4	31.7	0.2	121.2	0.8	41.6
18	0.38	31.8	1.9	53.6	3.6	46.1	0.7	30.3
19	0.18	38.6	6.1	31.9	ND	–	0.5	46.2
20	0.39	25.9	4.7	27.4	8.2	11.1	0.9	18.3
21	0.43	19.2	5.2	22.1	ND	–	1.1	25.9
22	0.34	26.7	5.4	22.4	9.6	26.7	1.6	29.0
23	0.45	21.6	4.6	35.1	9.1	17.4	1.4	34.5
24	0.50	12.9	4.2	33.0	7.0	20.6	1.4	22.4
25	0.43	37.6	4.4	34.5	5.9	25.5	1.5	51.6

*ND, Not Detected*.

To investigate the proportions of variance between plant and within plant, a fully nested ANOVA was undertaken, summarized in Table 3. For ergovaline and ergine, the main contributor of variance was variation in expression between plants. Chanoclavine showed the opposite pattern, with the main contributor being variation in expression within a plant. Lysergyl-alanine showed a more balanced distribution of variance.

**Table 3:** **Percentage of variance component attributed to between plant or within plant variation for each ergot alkaloid**.

**Alkaloid**	**Variance attributed to**	
	**Between plant (%)**	**Within plant (%)**
Chanoclavine	38.7	61.3
Ergovaline	70.9	29.1
Ergine	79.6	20.4
Lysergyl-alanine	43.5	56.5

**Figure 2:**
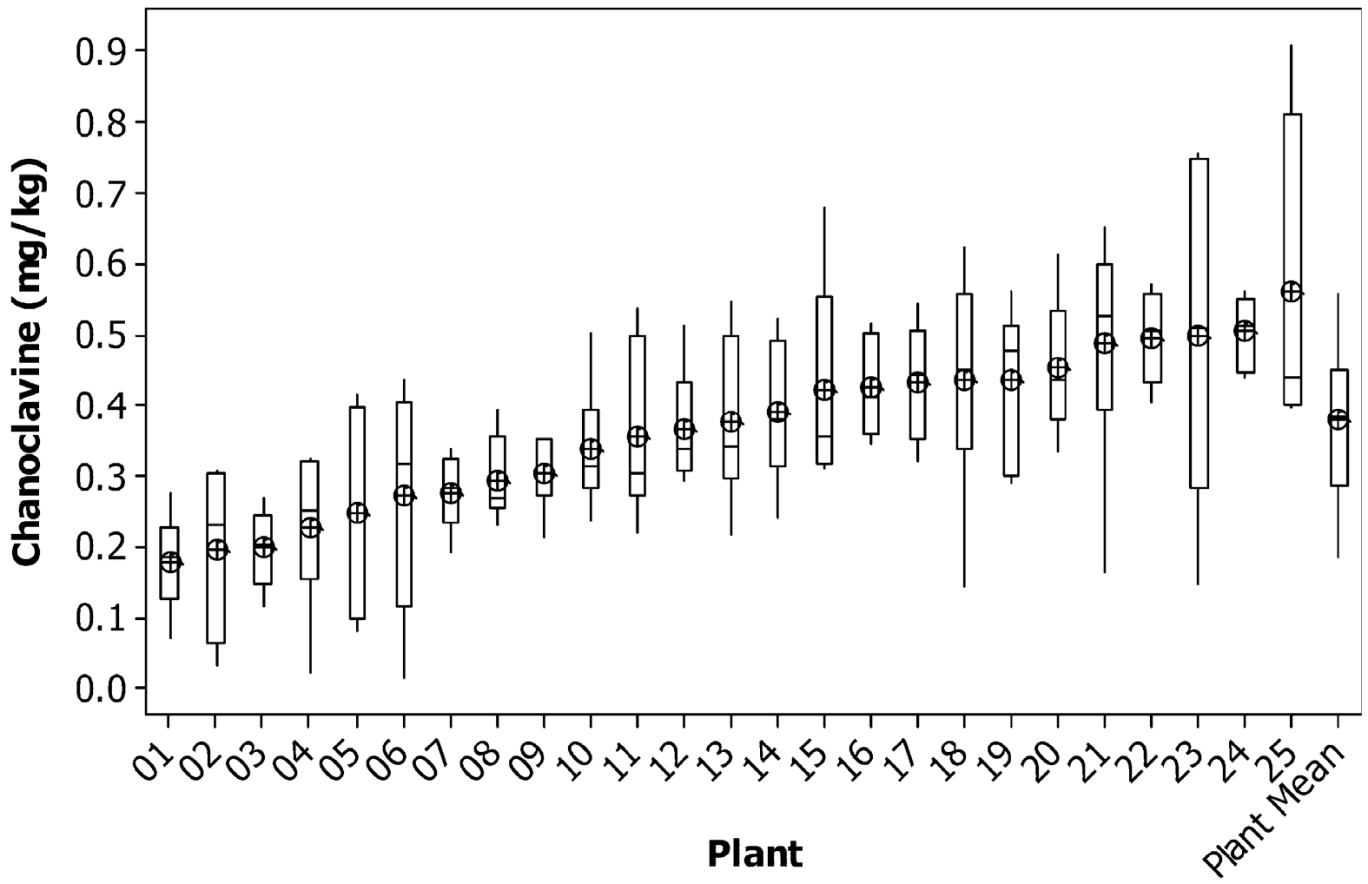
**Box plot of chanoclavine concentration for individual tillers of each plant with mean tiller concentration (open circles) and boxes showing interquartile range**. Plants are ranked from lowest to highest mean tiller concentration. Also shown are the corresponding parameters for the weighted mean of each plant.

**Figure 3:**
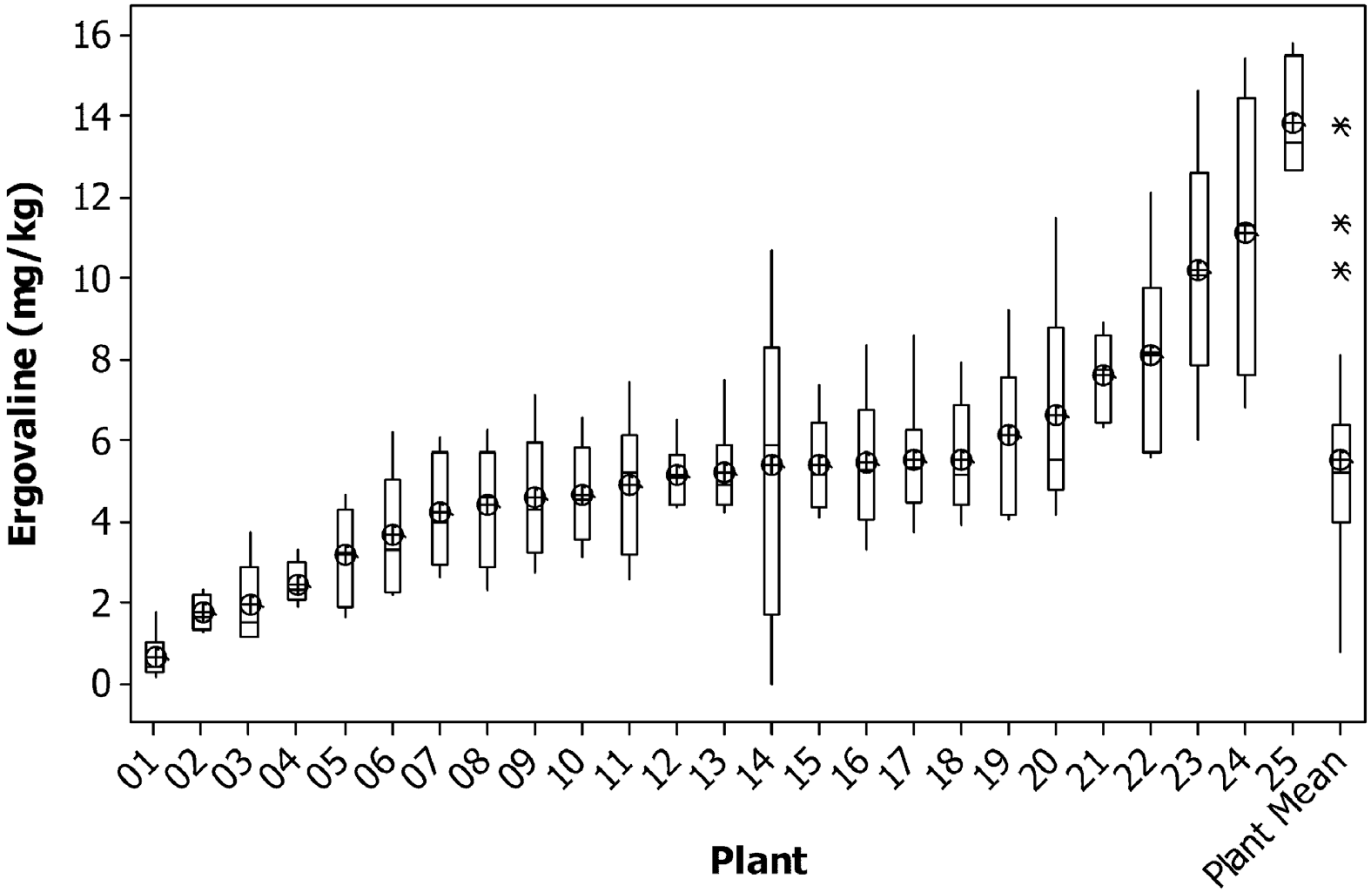
**Box plot of ergovaline concentration for individual tillers of each plant with mean tiller concentration (open circles) and boxes showing interquartile range**. Plants are ranked from lowest to highest mean tiller concentration. Also shown are the corresponding parameters for the weighted mean of each plant. Outliers are indicated by an asterisk.

For chanoclavine, as the concentration increased within a plant, the variance remained relatively constant. The average variance of the lowest five plants was similar to that of the highest five plants. This can be seen in the consistency of the size of the confidence intervals of the means of each plant (Figure 2). This compares to ergovaline, where the variance is greater for the higher expressing plants than for the lower expressing plants (Figure 3).

Using the data from the current study, power tables have been generated to determine the number of tillers that would be required to be harvested per plant to be confident in selecting the highest or lowest expressing chanoclavine or ergovaline plants of a population (Tables 4, 5, 6). From these power tables we can determine that it is not possible to select a set of low or high expressing chanoclavine individuals with 12 or fewer tillers, as the population mean is 0.37 ppm, with the lowest (0.18 ppm) and highest (0.56 ppm) expressing plants expressing within 0.2 ppm of the population mean. However, for ergovaline, it is possible to make selections within the population using relatively few tillers. Due to the smaller standard deviation at the lower concentration levels, it is possible that a sample of four tillers per plant will allow selection of a sub-set of plants with a mean 3 ppm lower than the population mean (equivalent to the bottom 20% of plants in the test population). To do the same selection for a sub-set of plants with a mean equivalent to the top 20% of plants in the test population (4.7 ppm greater than the population mean) would require sampling of more tillers (due to the higher standard deviation), but could still be achieved with 12 tillers per plant for a selection of 25 plants.

**Table 4:** **Power table indicating the number of tillers required to select a population of plants with indicated difference in mean chanoclavine expression (mg/kg) from the population mean**.

**Number of tillers**	**Plants in selection**				
	**5**	**10**	**15**	**20**	**25**
2	0.82	0.85	0.88	0.91	0.94
4	0.43	0.48	0.51	0.54	0.56
6	0.33	0.37	0.40	0.42	0.44
8	0.28	0.31	0.34	0.36	0.37
10	0.24	0.28	0.30	0.32	0.33
12	0.22	0.25	0.27	0.29	0.30

*Calculated for a 5% level of significance and a power of 0.8 using the standard deviation of the study population (0.15)*.

**Table 5:** **Power table indicating the number of tillers required to select a population of plants with indicated difference in mean ergovaline expression (mg/kg) from the population mean**.

**Tillers harvested**	**Plants in selection**				
	**5**	**10**	**15**	**20**	**25**
2	4.2	4.4	4.5	4.7	4.8
4	2.2	2.5	2.6	2.8	2.9
6	1.7	1.9	2.1	2.2	2.3
8	1.4	1.6	1.7	1.8	1.9
10	1.3	1.4	1.5	1.6	1.7
12	1.1	1.3	1.4	1.5	1.5

*Calculated for a 5% level of significance and a power of 0.8 using the standard deviation of plants with the lowest 20% expression within the study population (0.77)*.

**Table 6:** **Power table indicating the number of tillers required to select a population of plants with indicated difference in mean ergovaline expression (mg/kg) from the population mean**.

**Number of tillers**	**Plants in selection**				
	**5**	**10**	**15**	**20**	**25**
2	12.5	12.8	13.3	13.8	14.2
4	6.5	7.2	7.8	8.2	8.5
6	5.0	5.6	6.0	6.4	6.7
8	4.2	4.8	5.1	5.4	5.7
10	3.7	4.2	4.5	4.8	5.0
12	3.3	3.8	4.1	4.3	4.5

*Calculated for a 5% level of significance and a power of 0.8 using the standard deviation of plants with the highest 20% expression within the study population (2.27)*.

## Discussion

It has long been understood that there is significant variation in endophyte alkaloid expression between plants in a population (Easton et al., 2002). It is generally accepted that this is driven by the host genetics, and is a heritable response that can be selected for in a plant breeding program. What is not clearly understood is how much variation in endophyte alkaloid expression there is between tillers within a plant. This can have implications to plant breeding (through accuracy of plant selections) and in experimental design when investigating endophyte alkaloid expression, where it is necessary to ensure sufficient sample sizes to generate data that is statistically robust.

The results of this study have shown that it is important to not only have an understanding of the variation in the expression of endophyte alkaloids between plants in a population, but also the variation within the plants of the population. Unless the whole plant is being sampled (which is not always possible depending on the experimental design) the variation within the plants can have an impact of the quality of the data generated and the final conclusions of the study. Hence the variability within plants should be considered as part of the experimental design process.

### Conflict of interest statement

The authors declare that the research was conducted in the absence of any commercial or financial relationships that could be construed as a potential conflict of interest.
